# INTERLEUKIN-6 DRIVES MITOCHONDRIAL DYSREGULATION AND ACCELERATES PHYSICAL DECLINE: INSIGHTS FROM AN INDUCIBLE HUMANIZED IL-6 KNOCK-IN MOUSE MODEL

**DOI:** 10.1093/geroni/igae098.0568

**Published:** 2024-12-31

**Authors:** Peter Abadir

**Affiliations:** Johns Hopkins University, Baltimore, Maryland, United States

## Abstract

TBD

